# Ensembles of Convolutional Neural Networks and Transformers for Polyp Segmentation

**DOI:** 10.3390/s23104688

**Published:** 2023-05-12

**Authors:** Loris Nanni, Carlo Fantozzi, Andrea Loreggia, Alessandra Lumini

**Affiliations:** 1 Department of Information Engineering, University of Padova, 35122 Padova, Italy; carlo.fantozzi@unipd.it; 2 Department of Information Engineering, University of Brescia, 25121 Brescia, Italy; 3 Department of Computer Science and Engineering, University of Bologna, 40126 Bologna, Italy

**Keywords:** polyp segmentation, computer vision, ensemble, transformers, convolutional neural networks

## Abstract

In the realm of computer vision, semantic segmentation is the task of recognizing objects in images at the pixel level. This is done by performing a classification of each pixel. The task is complex and requires sophisticated skills and knowledge about the context to identify objects’ boundaries. The importance of semantic segmentation in many domains is undisputed. In medical diagnostics, it simplifies the early detection of pathologies, thus mitigating the possible consequences. In this work, we provide a review of the literature on deep ensemble learning models for polyp segmentation and develop new ensembles based on convolutional neural networks and transformers. The development of an effective ensemble entails ensuring diversity between its components. To this end, we combined different models (HarDNet-MSEG, Polyp-PVT, and HSNet) trained with different data augmentation techniques, optimization methods, and learning rates, which we experimentally demonstrate to be useful to form a better ensemble. Most importantly, we introduce a new method to obtain the segmentation mask by averaging intermediate masks after the sigmoid layer. In our extensive experimental evaluation, the average performance of the proposed ensembles over five prominent datasets beat any other solution that we know of. Furthermore, the ensembles also performed better than the state-of-the-art on two of the five datasets, when individually considered, without having been specifically trained for them.

## 1. Introduction

**Figure 1:**
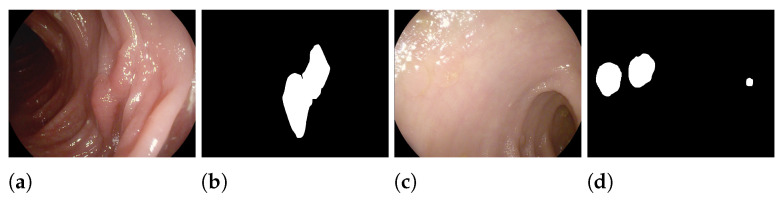
Two examples of the content of the ETIS-Larib [4] dataset for the semantic segmentation of polyps: (**a**,**c**) original images; (**b**,**d**) ground truth.

Colon polyps are among the preliminary manifestations of colorectal cancer, one of the cancers with the highest incidence [1]. The identification of precancerous polyps is essential during screening, as early detection and accurate diagnosis are the keys to effective treatment and low mortality [2]. Each 1% increase in polyp detection reduces the incidence of colon cancer by approximately 3% [3]. Currently, colonoscopy is the gold standard adopted in clinical practice to detect diseased tissue in the gastrointestinal tract. However, the accuracy of the actual match depends on the physician’s skill and requires a great deal of effort. Therefore, for the clinical prevention of colorectal cancer, it is crucial to have automatic methods that can point out all existing polyps with high accuracy. Artificial intelligence and machine learning models have been widely applied to the semantic segmentation of polyps in medical images. Two examples of colonoscopy images of polyps and their segmentations are shown in Figure 1.

Traditional approaches to segmentation have used, for example, geometric analysis and a frame-based model [5] or a hybrid context–shape approach [6]. Such approaches are hardly capable of extracting global context information and are not robust in complex scenes, mainly because they rely on hand-crafted features [7]. Deep learning has brought remarkable progress in the field of semantic segmentation: we refer the reader to [8] for a modern survey on the topic. Recently, deep networks have been applied to automatic polyp segmentation in colonoscopy images [9,10,11].

Regardless of the approach, the well-known “no free lunch” theorem for machine learning highlights that there cannot be a single model that works well on all datasets. Based on this evidence, an effective procedure is to adopt sets (ensembles) of classifiers, often shallow or weak, whose predictions are aggregated to form the output of the system. In an ensemble, individual classifiers are trained so that each generalizes differently in the training space. Ensembles provide state-of-the-art results in many domains, but it is important to secure some properties. One of them is to enforce some kind of diversity in the set of classifiers.

In this scenario, with this work, we provide two main contributions.

- An up-to-date review of ensembles for polyp segmentation. Actually, to the best of our knowledge, no earlier review on the topic is available in the open literature.
- A new ensemble for semantic segmentation based on the HarDNet-MSEG [12], Polyp-PVT [13], and HSNet [14] network topologies. The empirical evaluation showed that the performance for polyp segmentation was better than the state-of-the-art.

The idea behind the ensemble is to smooth the contribution of the fine-tuning of the hyperparameters for a specific dataset while averaging the performance of a model to deal with multiple domains [15]. We remark that the common practice of using an activation function, such as sigmoid, as the final layer of the model, followed by normalization, actually goes against this purpose because it can cause the average or sum rule to become too similar to a voting rule, thereby reducing the benefits of ensemble design. On the contrary, our ensemble is scientifically relevant because it introduces an approach to obtain smoother intermediate masks that are more suitable to be aggregated for the final segmentation. In turn, this approach shows that a better way to add transformers (part of Polyp-PVT and HSNet, tested in this work) to an ensemble is to modify the way the final segmentation mask is obtained. The ensemble also provides evidence that applying different approaches to the learning rate strategy is a viable method to build a set of segmentation networks. Furthermore, we demonstrate that a fusion of different convolutional and transformer topologies can achieve state-of-the-art performance.

The paper is organized as follows. Section 2 contributes a review of the literature on ensembles for polyp segmentation. Full details about our ensemble are provided in Section 3. Section 4 illustrates the results of our experiments and provides a comparison with the state-of-the-art. The paper concludes with Section 5, which contains some final remarks and outlines some research opportunities for the future.

## 2. Related Work

Several researchers have faced the problems of automatic polyp detection and segmentation. As in other domains, early attempts (e.g., [7]) focused on detection and were based on features extracted manually. More recently, deep networks have been applied to tasks, and interest has shifted toward segmentation [9,10,11,16]. Deep networks, together with other techniques [17] (Section B), are also in use for image enhancement [18]. In recent years, encoder–decoder architectures have gained popularity, with the most-widely adopted network in this family being U-Net [19]. Other popular networks adopted for polyp segmentation are, reportedly [20], DeepLabv3+, SegNet, FCNs, DeconvNet, PSPNet, and Mask R-CNN. All of them have been proposed in other domains. Several public datasets are available to researchers to train their networks and compare the performance. A recent summary of such datasets can be found in [21]. Details about the datasets we used in our experiments are provided in Section 3.3. In addition to segmentation accuracy, commonly measured by the Dice coefficient and the intersection over union (IoU) (definitions provided in Section 3.2), a performance metric considered by some works is segmentation time because the ultimate goal is to segment all frames of a colonoscopy video to identify as many precancerous polyps as possible and complete the job as fast as possible. All authors customarily report the mean value of the metrics over all the images in the test set, which is the same approach we followed in this paper.

It is essential to mention that state-of-the-art results are obtained by resorting to methods that increase performance beyond the level attained by baseline networks. Two methods are almost universally adopted in the realm of polyp detection and segmentation, as well as in other medical and nonmedical domains: data augmentation and ensemble techniques:

- Data augmentation [22] increases the size of the training set by adding synthetic samples. Such samples can be created in many ways: the most-common approach in computer vision is to generate new images by simply altering existing ones, for instance by flipping, cropping, or rotating them. However, other approaches are possible, including the generation of completely artificial images [23].
- Ensemble techniques [24] increase accuracy by combining the responses of different classifiers (per-pixel classifiers, in the case of semantic segmentation). As in the case of data augmentation, many different solutions have been proposed to combine the answers and to build the classifiers themselves.

Given the importance of ensemble techniques for this work, we now report on the literature on ensembles for polyp segmentation. To the best of our knowledge, 15 published works on the topic have been published. The salient features of these works are summarized in Table 1 (structure of the proposed ensembles), Table 2 (datasets used), Table 3 (performance metrics adopted), and Table 4 (reported performance). Among the 15 papers, we can report the following:

- Two [25,26] do not provide performance figures on public datasets. The source code is not available as well; hence, any comparison is beyond the bounds of possibility.
- Four [27,28,29,30] have been superseded by newer publications from the same authors, which show better performance on a wider range of datasets.
- Three [31,32,33] exhibit results that no longer represent the state-of-the-art for popular datasets, the more so considering that such results were obtained with more benevolent experimental protocols (e.g., ensembles trained and tested on the same dataset) than the one [10] currently adopted by several researchers, including ourselves.
- Four [20,34,35,36] report outstanding performance figures, but again, they were obtained with less stringent and/or incompletely documented protocols. Except for [36], the source code is not available.

The remaining two works [37,38] were included in the comparisons made during our experiments, as detailed in Section 4. We now briefly summarize, in chronological order, what we consider to be the most relevant of the 15 works on ensembles for polyp segmentation.

In [20], the polyp segmentation method was based on an ensemble built from three different networks: U-Net, SegNet, and PSPNet. The final segmentation was obtained through per-pixel weighted voting of the outputs of the three networks. The weights were proportional to the performance of the networks measured in the validation phase. Training, validation, and testing were performed with images from three public datasets: CVC-ColonDB [7] (300 images), CVC-ClinicDB [39] (612 images), and ETIS-Larib [4] (196 images). The training phase relied on transfer learning and data augmentation (scaling, flipping, rotations at different angles, and changes in brightness).

In [31], an ensemble of two Mask R-CNN models with different encoding backbones (ResNet-50 and ResNet-101 [40]) was proposed. The final segmentation was the bitwise combination, using the union operator, of the outputs of the two subnetworks. Transfer learning was adopted: the networks were pre-trained on the COCO dataset and fine-tuned with images from the same three datasets considered in [20]. As in [20], data augmentation (scaling, flipping, cropping, padding, random rotations, random shearing, random Gaussian blurring, random contrast normalization, and random changes in brightness) was adopted during training. The authors stated that this was an effective tool for improving segmentation performance, confirming the common conclusion in the literature.

In [34], the authors addressed the segmentation of multiple anatomical structures, including polyps. The proposed segmentation ensemble combines three DeepLabv3+ [41] variants trained with images at different resolutions and with different dilation strides. The authors stated that the ensemble thus captures information at multiple scales. Furthermore, a novel loss function was adopted that is a combination of the cross-entropy loss and the Dice loss. The discussion seems to imply that the outputs of the three networks were combined with a simple max function, that is if any of the three networks says that a given pixel belongs to a polyp, then this is the final output. The ensemble was trained and tested with images from the CVC-ColonDB, CVC-ClinicDB, and ETIS-Larib datasets. The datasets were augmented with standard geometric alterations (reflection, random cropping, translation rotation), elastic distortions, contrast normalization, and boundary enhancement.

In [32], the segmentation network combines the predictions of two U-Net models with ResNet-34 [40] and EfficientNet B2 [42] as their backbones; the details of how the outputs of the two models are combined to provide the final segmentation were not reported. The networks were trained with transfer learning, with initial weights obtained from ImageNet and fine-tuning performed with the publicly available Kvasir-SEG dataset [43] (1000 images). The segmentation accuracy was then tested with 160 images from the MediEval 2020 challenge. As in the previous works, the training phase leveraged data augmentation (scaling, flipping, random rotations, affine transformations, elastic deformations, CutMix regularization, random changes in contrast, and addition of Gaussian noise). The authors observed that CutMix regularization alone, which substitutes a block of pixels in a training image with a random patch from another image in the training batch, increased the accuracy by up to 3% in the validation set.

In [35], different networks (namely, MobileNet, ResNet, and EfficientNet [42]) were first tested as the backbones of U-Net, finding that EfficientNet provided the highest performance. Then, a new ensemble was proposed that combines the segmentation results of two U-Nets with EfficientNet B4 and EfficientNet B5 as the backbones. The outputs of the networks are combined asymmetrically: the output of the second (i.e., the one based on EfficientNet B5) is taken into account for a pixel only if its confidence level that such a pixel belongs to a polyp is greater than 0.96. A novel loss function was adopted during training to take into account the fact that the data were unbalanced, that is the number of non-polyp pixels is much higher than that of polyp pixels. The proposed function is a combination of the standard cross-entropy and asymmetric $F_{\beta }$ loss functions. As in all the papers mentioned, data augmentation (random scaling, cropping, padding, flipping, random rotations, random shearing, Gaussian blurring, random contrast normalization, and random changes in brightness) was employed during training. Transfer learning was also applied: the initial U-Net model was pre-trained on the ImageNet dataset and fine-tuned on the CVC-ClinicDB dataset. The performance of the ensemble was evaluated on the CVC-ColonDB and ETIS-Larib datasets.

Finally, we briefly mention [44], since the authors used the term “ensemble” to describe the networks they examined. However, what they did was test the variations of the U-Net architecture with different encoders. Each tested model was a single network, not an ensemble, with a different feature extractor. Based on the results of the experiments, the best-performing feature extractors were DenseNet169 and InceptionResNetV2. As in several other studies, extensive data augmentation (flipping, blurring, sharpening, random changes in contrast and brightness) was applied during training.

All the aforementioned works combined the outputs of two or three networks. A recent line of research has been the exploration of the accuracy advantages of bigger ensembles, whose predictions may be combined hierarchically or in some other complex fashion. In [36], two different ensembles for the semantic segmentation of polyps were discussed. The first ensemble, named TriUNet by the authors, combines three U-Net networks. The second ensemble, called DivergentNets, combines TriUNet with UNet++ [45], FPN [46], DeepLabv3, and DeepLabv3+. The final segmentation mask was an average of the five masks provided by these networks. DivergentNets can be considered an ensemble of size eight, albeit the outputs of the three networks were pre-combined in TriUNet. In [29], several ensembles were tested whose components differed in the backbones adopted, the loss functions, and the optimizers used in the training phase. The base architectures for the networks that made up the ensembles were DeepLabv3+ and HarDNet-MSEG [12]. The size of the ensembles ranged from 2 to 60. The ensembles were trained on 1450 images taken from the Kvasir-SEG and CVC-ClinicDB datasets, then tested on the remaining images from the same datasets (100 from Kvasir-SEG, 62 from CVC-ClinicDB), as well as on three “unseen” datasets: CVC-ColonDB, ETIS-Larib, and the test set from CVC-EndoSceneStill [47]. This is an experimental protocol that was first introduced in [10]. In [37], the authors proposed ensembles of the DeepLabv3+, HarDNet-MSEG, and Polyp-PVT [13] networks, trained with different loss functions and data augmentation methods. A wider range of loss functions was considered than in [29], including weighted combinations of base functions, and more than ten data augmentation techniques were applied in the training phase. The size of the ensembles ranged from 2 to 14. The datasets used for training and testing were the same as in [10]. In [26], an ensemble was proposed that combines the predictions of Eff-UNet [48], nnU-Net [49] and a hierarchical multiscale attention network [50]. The training set was the one provided by the EndoCV2022 polyp segmentation sub-challenge, with the addition of images from CVC-ColonDB, CVC-ClinicDB, and ETIS-Larib. The training set was manually curated by the authors to remove images with implausible annotations; it is not publicly available. Moreover, the only performance data provided by the authors were on “folds” of data that do not have a documented relationship with public datasets. In [38], the ensemble was made up of two different sub-ensembles, once again based on DeepLabv3+ (backbone: ResNet-101) and HarDNet-MSEG (backbone: HarDNet-68), respectively. Inside each sub-ensemble, diversity was provided by varying the activation functions (15 different loss functions were considered) and the data augmentation strategies. Additionally, in the sub-ensemble based on DeepLabv3+, polyps identified by different networks were not allowed to overlap. The training and testing protocols were, once again, those introduced in [10].

**Table 1:** Ensembles for polyp segmentation: structure of the ensembles. The paper [44] does not appear in this table and in Table 2. The authors used the term ensemble to describe the networks they examined. However, what they did was test variations of the U-Net architecture with different encoders.

Paper	Ensemble Size	Deep Labv3	Deep Labv3+	Eff- UNet	FPN	HarDNet- MSEG	Mask R-CNN	MultiRes UNet	nnU-Net	PSPNet	Polyp- PVT	SegNet	U-Net	Unet++
Guo2019 [20]	3									🗸		🗸	🗸	
Kang2019 [31]	2						🗸							
Nguyen2019 [34]	3		🗸											
Shrestha2020 [32]	2												🗸	
ThuHong2020 [35]	2												🗸	
Hong2021 [33]	5						🗸							
Lumini2021a [27]	14		🗸											
Lumini2021b [28]	14		🗸											
Nanni2021 [29]	2, 10, 20, 30, 60		🗸			🗸								
Thambawita2021 [36]	3, 7	🗸	🗸		🗸								🗸	🗸
Tomar2021 [25]	4							🗸						
Nanni2022a [37]	2, 10, 14		🗸			🗸					🗸			
Nanni2022b [30]	12, 14, 15, 16, 20		🗸			🗸								
Tran2022 [26]	N/A			🗸					🗸					
Nanni2023 [38]	2, 4, 10, 20, 30		🗸			🗸								

**Table 2:** Ensembles for polyp segmentation: datasets used. Some names are abbreviated; see Section 3.3. Datasets can be used for training, testing, or both. Datasets from other domains used as a consequence of transfer learning are not listed.

Paper	ColDB	ClinDB	CVC-T	EDD 2020	Polyp-Gen	ETIS	Hyper-Kvasir	Kvasir	MediEval 2020
Guo2019 [20]	🗸	🗸				🗸			
Kang2019 [31]	🗸	🗸				🗸			
Nguyen2019 [34]	🗸	🗸				🗸			
Shrestha2020 [32]								🗸	🗸
ThuHong2020 [35]	🗸	🗸				🗸			
Hong2021 [33]					🗸				
Lumini2021a [27]								🗸	
Lumini2021b [28]								🗸	
Nanni2021 [29]	🗸	🗸	🗸			🗸		🗸	
Thambawita2021 [36]					🗸		🗸		
Tomar2021 [25]					🗸				
Nanni2022a [37]	🗸	🗸	🗸			🗸		🗸	
Nanni2022b [30]	🗸	🗸	🗸			🗸		🗸	
Tran2022 [26]	🗸	🗸		🗸	🗸	🗸			
Nanni2023 [38]	🗸	🗸	🗸			🗸		🗸	

**Table 3:** Ensembles for polyp segmentation: performance metrics. “FPS” stands for “frames per second”. The authors of [25] provided only a score specific to the EndoCV 2021 Segmentation Generalization Challenge, named “generalization score”.

Paper	Accuracy	Dice	F2	FPS	IoU	Precision	Recall	Sensitivity	Specificity
Guo2019 [20]		🗸			🗸				
Kang2019 [31]					🗸	🗸	🗸		
Nguyen2019 [34]	🗸	🗸				🗸	🗸	🗸	🗸
Shrestha2020 [32]	🗸	🗸	🗸	🗸	🗸	🗸	🗸		
ThuHong2020 [35]		🗸			🗸	🗸	🗸		
Hong2021 [33]	🗸	🗸	🗸	🗸	🗸	🗸	🗸		
Lumini2021a [27]	🗸	🗸	🗸		🗸	🗸	🗸		
Lumini2021b [28]	🗸	🗸	🗸		🗸	🗸	🗸		
Nanni2021 [29]		🗸			🗸				
Thambawita2021 [36]		🗸			🗸	🗸	🗸		
Tomar2021 [25]									
Nanni2022a [37]		🗸			🗸				
Nanni2022b [30]	🗸	🗸	🗸		🗸	🗸	🗸		
Tran2022 [26]		🗸							
Nanni2023 [38]		🗸			🗸				

**Table 4:** Ensembles for polyp segmentation: performance (Dice coefficient and IoU) reported by the authors, rounded to three significant digits. For papers that propose more than one ensemble, the table reports the figures for the best ensemble. Papers and datasets for which no scores are available do not appear in the table. Note that the results from different papers are, in general, not comparable because they were obtained with different testing protocols.

Paper	Kvasir		ClinDB		ColDB		ETIS		CVC-T		PolypGen		MediEval 2020	
	IoU	Dice	IoU	Dice	IoU	Dice	IoU	Dice	IoU	Dice	IoU	Dice	IoU	Dice
Guo2019 [20]			0.967	0.983	0.962	0.980	0.970	0.985						
Kang2019 [31]					0.695		0.661							
Nguyen2019 [34]				0.891		0.896								
Shrestha2020 [32]	0.760	0.838											0.755	0.832
ThuHong2020 [35]					0.798	0.891	0.702	0.823						
Hong2021 [33]											0.571	0.619		
Lumini2021a [27]	0.820	0.885												
Lumini2021b [28]	0.825	0.888												
Nanni2021 [29]	0.872	0.919	0.886	0.931	0.701	0.776	0.663	0.743	0.831	0.901				
Thambawita2021 [36]											0.840			
Nanni2022a [37]	0.874	0.920	0.894	0.937	0.751	0.826	0.717	0.787	0.842	0.904				
Nanni2022b [30]	0.870	0.918	0.884	0.929	0.695	0.768	0.644	0.727	0.833	0.904				
Nanni2023 [38]	0.871	0.920	0.903	0.947	0.720	0.787	0.688	0.756	0.846	0.909				

## 3. Methods

In this section, we introduce and describe the methods adopted in this work, as well as the datasets and the functions used to assess the performance.

### 3.1. Structure of the Ensemble

As anticipated in Section 1, our ensemble is based on the HarDNet-MSEG [12], Polyp-PVT [13], and HSNet [14] network topologies. These models were selected for the following reasons: HarDNet-MSEG is a lightweight neural network that has shown very good performance in the polyp segmentation task; Polyp-PVT is one of the first attempts to use transformers in the field of polyp segmentation; HSNet is a mixed model including both CNN and transformers in the encoder. Our final ensemble includes multiple instances of the three models, trained in different ways as summarized in Section 3.4 and described in full in Section 4. In our preliminary experiments, we observed that, in these models, the last sigmoid layer followed by a normalization layer goes against the purpose of ensemble design because it pushes the scores to the extremes of the range, making the average or sum rule too similar to a voting rule.

A novelty in our network architecture is that we cut the normalization layer after the last sigmoid in HarDNet-MSEG, Polyp-PVT, and HSNet. In the original networks, before the output, each segmentation mask is normalized between [0,1]: this implies that the networks always find a foreground object, but this assumption cannot be made in a real colonoscopy. Therefore, the reported results obtained using HarDNet-MSEG, Polyp-PVT, and HSNet are slightly different from the ones in the original papers.

**Figure 2:**
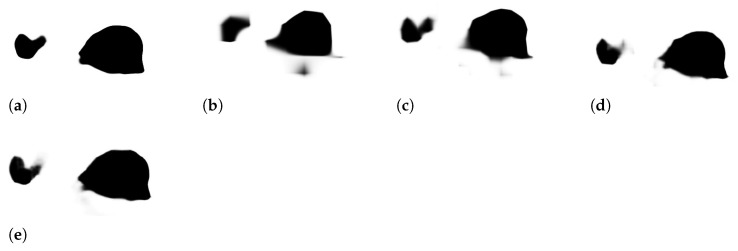
Masks in HSNet. (**a**) Final segmentation mask. (**b**–**e**) Intermediate masks after the sigmoid computation.

Another significant difference is that, while in the original Polyp-PVT and HSNet topologies, the intermediate masks (two masks for Polyp-PVT and four for HSNet) are summed and then passed to the sigmoid, we passed each mask separately to the sigmoid and averaged the results. Consequently, our output is given by

$$\sum_{i=1}^{n}sigmoid\left(P_{i}\right)/n,$$

where $P_{i}$ is an intermediate prediction mask and *n* is the number of the masks (n=2 for Polyp-PVT, n=4 for HSNet). In Figure 2, we compare the output of HSNet and the related four intermediate masks. It is clear that the output is sharper, so the sum rule is almost a voting rule if the original outputs of HSNet and Polyp-PVT are used in an ensemble.

### 3.2. Performance Metrics and Loss Functions

In this section, we summarize the performance metrics and the loss functions adopted in this paper. For an exhaustive overview of image segmentation and loss functions, we point the interested reader to the recent survey [51].

We adopted the Dice coefficient [52] to measure the overlap between the predicted segmentation masks and the ground truth. This approach is widespread in semantic segmentation. The Dice coefficient is defined as

$$\mathrm{Dice}\left(Y,T\right)=\frac{2\times |Y\cap T|}{\left|Y|+|T\right|},$$

where *Y* is the predicted segmentation mask, *T* is the ground-truth mask, and the cardinality is the number of pixels.

Another well-known performance measure is the intersection over union (IoU), which was introduced in [53]:

$$\mathrm{IoU}\left(Y,T\right)=\frac{\left|Y\cap T\right|}{\left|Y\cup T\right|}.$$

Hence, an IoU of 1 corresponds to a perfect prediction, that is a pixel-perfect overlap between the predicted segmentation mask and the ground truth. The corresponding loss function is defined as

$$L_{IoU}^{\prime }=1-IoU.$$

This could be an issue when dealing with imbalanced datasets. Therefore, as suggested in [54], we used the weighted intersection over union (wIoU) instead of the standard IoU. The corresponding loss function is

$$L_{wIoU}^{\prime }=1-\frac{1+\sum_{i=1}^{N}\sum_{k=1}^{K}w_{ik}T_{ik}*Y_{ik}}{1+\sum_{i=1}^{N}\sum_{k=1}^{K}w_{ik}\left(T_{ik}+Y_{ik}-T_{ik}*Y_{ik}\right)},$$

where *N* is the number of pixels, *K* is the number of classes, and $w_{ik}$ is the weight given to the *i*-th pixel of the image for the class *k*. These weights were computed as before. $T_{ik}$ and $Y_{ik}$ are, respectively, the ground truth value and the prediction value for the *i*-th pixel belonging to the class *k*. We added 1 to both the numerator and the denominator to prevent undefined divisions.

The cross-entropy (CE) loss function provides us with a measure of the difference between two probability distributions. The goal is to minimize this difference, and in doing so, it has no bias between small or large regions. This could be an issue when dealing with imbalanced datasets. Hence, the weighted CE loss was introduced, and it resulted in well-balanced classifiers for imbalanced scenarios [55]. The formula for the weighted binary CE loss is

$$L_{wBCE}=-\sum_{i=1}^{N}\sum_{k=1}^{K}w_{ik}T_{ik}*log\left(P_{ik}\right),$$

where *N* is the number of pixels, *K* is the number of classes, and $w_{ik}$ is the weight given to the *i*-th pixel of the image for the class *k*. These weights were computed by using an average pooling over the mask with a kernel of size 31×31 and a stride of 1 to consider also non-maximal activations. $T_{ik}$ is the true value for the *i*-th pixel, and it can be equal to either 0 or 1. It is 1 if the *i*-th pixel belongs to the class *k*, 0 otherwise. $P_{ik}$ is the probability that the *i*-th pixel belongs to the class *k* obtained by using the sigmoid activation function. For *P*, we used the softmax activation function, which returns probabilities.

Based on the intuition in [12], the wIoU loss and the weighted binary CE loss were considered together (structure loss function):

$$L_{STR}^{\prime }=L_{wIoU}+L_{wBCE}.$$

In our experiments, the structure loss function was used to train all the networks except DeepLabv3+.

### 3.3. Datasets and Data Augmentations

Polyp segmentation from colonoscopy images is a challenging task that requires two-class discrimination between polyp pixels and the low-contrast colon background. We present experimental results on five datasets for polyp segmentation:

- The Kvasir-SEG [56] dataset (“Kvasir”) contains medical images that have been labeled and verified by doctors. The images depict different parts of the digestive system and show both healthy and diseased tissue. The dataset includes images at different resolutions (from 720 × 576 up to 1920 × 1072 pixels) and is organized into folders based on the content of the images. Some of the images also include a small picture-in-picture showing the position of the endoscope inside the body.
- CVC-ColonDB [7] (“ColDB”) is a dataset of 300 images that aims to include a wide range of appearances for polyps. The goal is to provide as much diversity as possible in the dataset.
- CVC-T (sometimes called “Endo”) is the test set of a larger dataset named CVC-EndoSceneStill [47].
- The ETIS-Larib [4] dataset (“ETIS”) contains 196 colonoscopy images. Two frames from the dataset and their corresponding ground truth masks are shown in Figure 1.
- CVC-ClinicDB [39] (“ClinDB”) contains 612 images from 31 videos of colonoscopy procedures. The images have been manually labeled by experts to identify the regions covered by the polyps, and ground truth information is also provided for light reflections. The images are 576 × 768 pixels in size.

Our training set is made of 1450 images taken from the largest datasets, namely 900 images from Kvasir and 550 images from ClinDB. The remaining images (100 from Kvasir, 62 from ClinDB, plus all the images from ColDB, CVC-T, and ETIS) were used for the test set. As pointed out in Section 2, this experimental protocol was proposed in [10] and has been followed by many, including the researchers behind the three base networks we considered (see Section 3.1). Having a small training set is a common challenge in deep learning and can often lead to overfitting, i.e., the model memorizes the training data rather than learning generalizable patterns. In this work, we adopted two common techniques for addressing this issue: fine-tuning and data augmentation. Fine-tuning was used since all the models involved in our experiments were pre-trained on a large dataset. Data augmentation is based on two different strategies, which aim to increase the effective size of the training set and provide the model with more examples to learn from:

- “DA1”: a basic strategy that includes two image flips (up/down and left/right) and a 90-degree counterclockwise rotation; therefore, three synthetic images were created for each original image.
- “DA2”: a sophisticated strategy that creates synthetic images in 13 different ways, including the application of motion blur and shadows to the original images.

**Figure 3:**
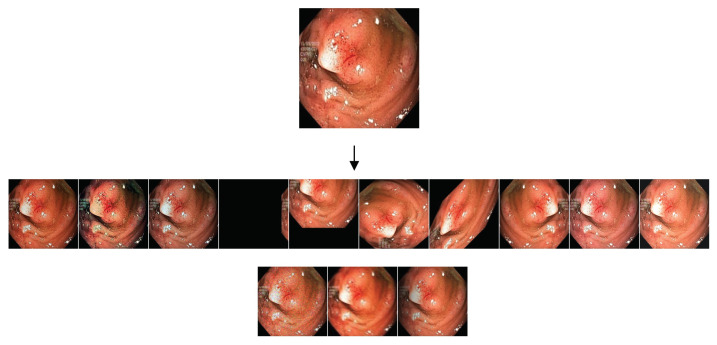
An example of images obtained through our data augmentation process. (**Top**) Original polyp image. (**Bottom**) Synthetic images created from the original image by data augmentation.

The two strategies were introduced in [37]; we refer the interested reader to this work for a thorough description. Figure 3 highlights the images provided by the data augmentation process for a sample polyp image.

### 3.4. Overview of the Experiments

**Figure 4:**
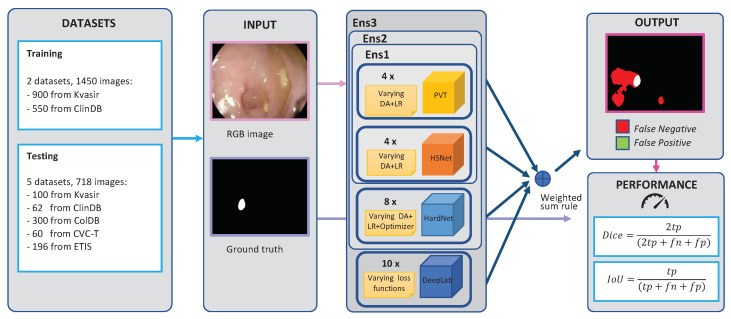
Overall structure of the proposed method, including the training and testing procedures.

In our experiments, we considered different ensembles built with the base networks HarDNet-MSEG, Polyp-PVT, and HSNet (see Section 3.1). Polyp-PVT and HSNet are based on transformers, introduced recently [57] in the domain of polyp segmentation. Figure 4 shows the overall structure of the network for the most-complex ensemble we investigated in the experimental section (Section 4, Ensemble Ens3). The networks were trained for 100 epochs with a batch size of 20 for HarDNet-MSEG and 8 for Polyp-PVT and HSNet. To avoid any overfitting, we used the default value for the number of epochs. The networks were always trained using the structure loss function (see Section 3.2) except for DeepLabv3+, and their outputs were combined with the sum rule or the weighted sum rule depending on the experiment. We used resized training images of size 352 × 352. During the test phase, masks were obtained by resizing the images and were subsequently scaled back to the original dimensions to evaluate the performance of the model.

As optimization methods, we experimented with both Adam and stochastic gradient descent (SGD) for HarDNet-MSEG, while we adopted AdamW for Polyp-PVT and HSNet, like in the original papers.

We trained the networks with the two data augmentation techniques described in Section 3.3. We also experimented with training two identical networks with the two techniques and combining their outputs.

Finally, the following two learning rates were considered in the experiments:

- $10^{-4}$ (learning rate “a”);
- $5\times 10^{-5}$ decaying to $5\times 10^{-6}$ after 30 epochs (learning rate “b”).

We also trained some networks twice with the two learning rates and, as we did with the data augmentations, combined their outputs.

## 4. Experimental Results

In this section, we report on the experimental analysis carried out to assess the proposed ensemble strategy.

### 4.1. Ablation Studies

Ablation in machine learning refers to removing parts of a machine learning model to measure their impact on the performance. In this work, we employed an ensemble approach by combining the predictions of the three models to improve the overall performance of our system. To evaluate the contributions of the different components in our ensemble, we conducted an ablation study by separately analyzing the potential of each model. The results are summarized in Table 5, Table 6 and Table 7, respectively. Note that the performance of the ensembles using multiple models will be described later, in Section 4.2. The Dice coefficient was used to measure the performance on the five test sets defined in Section 3.3. In the column DA, we specify which data augmentation approaches (see Section 3.3) were considered. Where the column states “1 + 2”, we trained the network twice with Data Augmentations 1 and 2. In the column LR, we specify which learning rates (see Section 3.4) were used during training. Where the column states “a + b”, we trained the network twice with Learning Rates a and b. In Table 5, the additional column OPT specifies which optimization methods (see Section 3.4) were adopted:

- “SGD”: stochastic gradient descent.
- “Adam”: Adam.
- “SGD + Adam”: both.

If a network was trained multiple times with different choices, the outputs were combined with the sum rule, and the final model was, therefore, an ensemble. Consequently, the last three rows in the table represent ensembles of 4, 4, and 8 HarDNet-MSEG networks, respectively.

The conclusions we can draw from the results in Table 5 are that, on average, Learning Rate a provided better results than Learning Rate b. Learning Rate b appeared to perform poorly when coupled with SGD. The best average performance was provided by the ensemble that adopts both different data augmentation strategies and different learning rates (last row of Table 5); however, the Dice coefficient was only marginally better than that of the ensemble trained with Learning Rate a alone (Row 9).

In Table 6 and Table 7, the additional column SM specifies whether we obtained the final segmentation mask as in the original HSNet and Polyp-PVT networks (SM = “No”) or with the novel approach we proposed in Section 3.1 (SM = “Yes”).

The conclusions we can draw from the results reported in Table 6 and Table 7 are that, on average, Learning Rate b performed better than Learning Rate a when coupled with HSNet and slightly better when coupled with Polyp-PVT. For both Polyp-PVT and HSNet, the best average performance was obtained by the ensemble obtained by varying the data augmentation and the learning rate.

**Figure 5:**
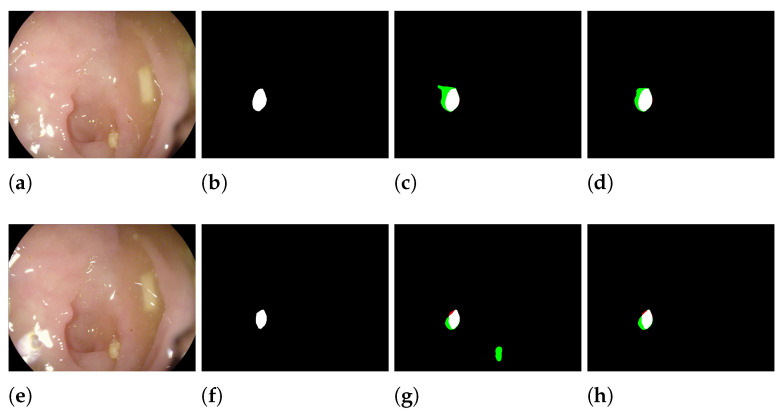
Masks in HSNet ensemble with and without smoothing. (**a**,**e**) Original image, (**b**,**f**) ground truth, (**c**,**g**) HSNet ensemble without smoothing, and (**d**,**h**) HSNet ensemble with smoothing. False positive pixels are green. False negative pixels are red.

Using the proposed approach to obtain the segmentation masks allowed us to increase the performance of the Polyp-PVT/HSNet ensembles. Some inference masks obtained using HSNet ensembles (HSNet a + b) with and without smoothing are reported in Figure 5, where false positive pixels are highlighted in green, while the false negatives are in red. They demonstrate that our ensemble model with smoothing produced better boundary results and made more accurate predictions than the models without smoothing.

In Table 8, we report the output values before the sigmoid layer for HarDNet-MSEG and the intermediate masks of Polyp-PVT and HSNet. All the networks were trained with Data Augmentation 1 and Learning Rate a. In the last two columns, the non-saturation rate of the resulting masks is reported using our averaging rule, compared to the original sum rule followed by normalization. Non-saturation is measured as the percentage *p* of pixels such that abs(p)<6.9 (since sigmoid(6.9)≈0.999). It can be observed that these output values are all very close to saturating the sigmoid function. Summing them together would exacerbate the situation by producing an almost binary output. As shown in the last two columns, our approach has a higher rate of non-saturating pixels in the sigmoid function. Our approach averages the intermediate masks and produces a smoother output, which means that it maintains more information. This information can be visually appreciated in Figure 5. In particular, it can be noticed that the final segmentation mask of HSNet (Figure 5a) is very sharp, indicating an almost binary output, while the four intermediate masks $P_{1}$–$P_{4}$ have more blurred edges.

### 4.2. Proposed Ensembles and Comparison with State-of-the-Art Models

In this section, we compared the performance of the three ensembles proposed in this work with twenty state-of-the-art methods for polyp segmentation (Table 9). In our final proposed ensembles, the models analyzed in Section 4.1 were combined with the weighted sum rule. Each method was weighted so that its weight in the fusion is equal to the other methods. We report the performance of the following ensembles:

- “Ens1”: an ensemble of 4 Polyp-PVT networks with the segmentation masks obtained with our approach and trained with all possible combinations of Data Augmentations 1 and 2 and Learning Rates a and b (Table 6, last row), plus 4 HSNet networks with the segmentation masks obtained with our approach and trained with all possible combinations of Data Augmentations 1 and 2 and Learning Rates a and b (Table 7, last row).
- “Ens2”: like Ens1, plus eight HarDNet-MSEG networks trained with all possible combinations of the SGD and Adam optimizers, Data Augmentations 1 and 2, and Learning Rates a and b (Table 5, last row).
- “Ens3”: like Ens2, plus the best ensemble introduced in [37] based on DeepLabv3+.

From the results reported in Table 9, it can be seen that the proposed ensembles beat the state-of-the-art on the ColDB and ETIS datasets, which can be regarded as the most-challenging since the corresponding Dice scores were consistently lower not only for the ensemble components and ensembles (Table 5, Table 6 and Table 7 and Table 9, Rows 1–3), but also for competing solutions in the literature (Table 9, Rows 4–23). We remark that this result was obtained without training the ensembles on ColDB and ETIS. Most importantly, all three proposed ensembles performed better than state-of-the-art solutions when we averaged across all datasets (Table 9, last two columns). Even the simplest of our ensembles, that is Ens1, beat the state-of-the-art on average. The conclusion we can draw is that the proposed ensembles were strong performers with all the datasets: even when they were not the best, they were near the top. This is a benefit of the ensemble strategy. A change in performance with the dataset remains unavoidable: images in different datasets were obtained with different acquisition instruments, under different conditions, and created by experts with different opinions on what is important to include in the dataset. For instance, the authors of ColDB deemed it important to exclude similar frames from the dataset and, in general, to maximize the variability between polyps in different images. Again, this makes ColDB a challenging dataset. Of course, the three ensembles performed significantly better than their baseline networks (Table 9, Rows 4–7), including the recent ensemble introduced in [37]. The usefulness of transformers emerges clearly from the performance difference between Ens1 (transformers) and HarDNet-MSEG (best CNN tested). Polyp-PVT and HSNet were the best transformer-based methods; their fusion in Ens1 allowed for clearly better performance than that obtained by any of the two methods alone, as seen by comparing Ens1 with Polyp-PVT and HSNet. On the other hand, adding CNN-based methods to Ens1, as was done in Ens2 and Ens3, only led to marginal improvements.

**Table 9:** Performance comparison between the proposed ensembles and recent models available in the literature. The best performance figures are marked in bold. Underlined figures point out where our ensembles beat, or were on par with, the state-of-the-art.

Method	Kvasir		ClinDB		ColDB		ETIS		CVC-T		*Avg*	
	IoU	Dice	IoU	Dice	IoU	Dice	IoU	Dice	IoU	Dice	IoU	Dice
*Ens1*	**0.886**	0.930	0.892	0.936	**0.764**	**0.839**	0.738	0.812	0.841	0.904	0.824	0.884
*Ens2*	0.883	0.927	0.894	0.938	0.759	0.832	0.750	0.822	0.841	0.904	0.825	**0.885**
*Ens3*	0.882	0.928	0.894	0.937	0.752	0.824	**0.760**	**0.830**	0.840	0.905	**0.826**	**0.885**
HarDNet-MSEG [12]	0.857	0.912	0.882	0.932	0.66	0.731	0.613	0.677	0.821	0.887	0.767	0.828
Polyp-PVT [13]	0.864	0.917	0.889	0.937	0.727	0.808	0.706	0.787	0.833	0.9	0.804	0.869
HSNet [14]	0.877	0.926	**0.905**	**0.948**	0.735	0.81	0.734	0.808	0.839	0.903	0.818	0.879
FH(2)+2×PVT(2) [37]	0.874	0.920	0.894	0.937	0.751	0.826	0.717	0.787	0.842	0.904	0.816	0.875
PraNet (from [12])	0.84	0.898	0.849	0.899	0.64	0.709	0.567	0.628	0.797	0.871	0.739	0.801
SFA (from [12])	0.611	0.723	0.607	0.7	0.347	0.469	0.217	0.297	0.329	0.467	0.422	0.531
U-Net++ (from [12])	0.743	0.821	0.729	0.794	0.41	0.483	0.344	0.401	0.624	0.707	0.57	0.641
U-Net (from [12])	0.746	0.818	0.755	0.823	0.444	0.512	0.335	0.398	0.627	0.71	0.581	0.652
Eloss101-Mix + FH [38]	0.871	0.920	0.903	0.947	0.720	0.787	0.688	0.756	0.846	0.909	0.806	0.864
MIA-Net [58]	0.876	0.926	0.899	0.942	0.739	0.816	0.725	0.8	0.835	0.9	0.815	0.877
P2T [59]	0.849	0.905	0.873	0.923	0.68	0.761	0.631	0.7	0.805	0.879	0.768	0.834
DBMF [60]	**0.886**	**0.932**	0.886	0.933	0.73	0.803	0.711	0.79	**0.859**	**0.919**	0.814	0.875
SETR [61]	0.854	0.911	0.885	0.934	0.69	0.773	0.646	0.726	0.814	0.889	0.778	0.847
TransUnet [62]	0.857	0.913	0.887	0.935	0.699	0.781	0.66	0.731	0.824	0.893	0.785	0.851
TransFuse [11]	0.87	0.92	0.897	0.942	0.706	0.781	0.663	0.737	0.826	0.894	0.792	0.855
UACANet [63]	0.859	0.912	0.88	0.926	0.678	0.751	0.678	0.751	0.849	0.91	0.789	0.85
SANet [64]	0.847	0.904	0.859	0.916	0.67	0.753	0.654	0.75	0.815	0.888	0.769	0.842
MSNet [65]	0.862	0.907	0.879	0.921	0.678	0.755	0.664	0.719	0.807	0.869	0.778	0.834
SwinE-Net [66]	0.87	0.92	0.892	0.938	0.725	0.804	0.687	0.758	0.842	0.906	0.803	0.865
AMNet [67]	0.865	0.912	0.888	0.936	0.69	0.762	0.679	0.756	-	-	-	-

## 5. Conclusions

In this work, we provided a review of the literature on deep learning ensembles for polyp segmentation and demonstrated the advantages of tackling semantic segmentation with ensembles of convolutional and transformer neural networks. The main idea behind ensembling is to combine the predictions of multiple models to improve the overall performance. We introduced an effective way of doing this by averaging intermediate predictions in a new fashion. This can help to smooth out the contribution of any specific model, besides reducing the impact of overfitting to a particular dataset.

We plan to generalize our results to other application domains. For this reason, many datasets will be used in the future to corroborate the conclusions reported here, namely to prove that:

- A fusion of different convolutional and transformer topologies can achieve state-of-the-art performance;
- Applying different approaches to the learning rate strategy is a feasible method to build a set of segmentation networks;
- A better way to add the transformers (Polyp-PVT and HSNet) in an ensemble is to use the proposed approach for creating the final segmentation mask.

Furthermore, we plan to test our model with different distillation techniques and pruning approaches to adapt this technique to low-cost hardware. This will allow us to extend the usefulness of our model to situations where the available computational power is restricted or constrained.

## Figures and Tables

**Table 5:** Dice coefficient for the (ensemble) networks built on HarDNet-MSEG. The best performance figures are marked in bold.

OPT	DA	LR	Kvasir	ClinDB	ColDB	ETIS	CVC-T	*Avg*
SGD	1	a	0.893	0.875	0.745	0.667	0.882	0.812
		b	0.862	0.794	0.705	0.628	0.873	0.772
SGD	2	a	0.914	0.944	0.747	0.727	0.901	0.847
		b	0.871	0.875	0.702	0.650	0.885	0.797
Adam	1	a	0.906	0.924	0.751	0.716	0.903	0.840
		b	0.910	0.910	0.748	0.702	0.884	0.831
Adam	2	a	0.896	0.927	0.778	0.774	0.893	0.854
		b	0.895	0.916	0.758	0.716	0.885	0.834
SGD + Adam	1 + 2	a	**0.918**	**0.947**	0.778	0.756	**0.909**	0.862
		b	0.907	0.928	0.775	0.770	0.899	0.856
		a + b	0.915	0.931	**0.785**	**0.781**	0.904	**0.863**

**Table 6:** Dice coefficient for the (ensemble) networks built on Polyp-PVT. The best performance figures are marked in bold.

DA	LR	SM	Kvasir	ClinDB	ColDB	ETIS	CVC-T	*Avg*
1	a	No	0.911	0.926	0.788	0.773	0.871	0.854
1	b	No	0.924	0.924	0.793	0.800	0.877	0.864
2	a	No	0.910	0.923	0.804	0.749	0.891	0.855
2	b	No	0.917	0.921	0.794	0.763	0.891	0.857
1 + 2	a	No	0.918	0.926	0.803	0.755	0.873	0.855
1 + 2	a	Yes	0.919	0.930	0.809	0.765	0.884	0.861
1 + 2	b	No	**0.931**	0.920	0.792	0.776	0.876	0.859
1 + 2	b	Yes	**0.931**	0.921	0.798	0.791	0.882	0.865
1 + 2	a + b	No	0.926	0.932	0.821	0.800	0.891	0.874
1 + 2	a + b	Yes	0.926	**0.933**	**0.824**	**0.808**	**0.895**	**0.877**

**Table 7:** Dice coefficient for the (ensemble) networks built on HSNet. The best performance figures are marked in bold.

DA	LR	SM	Kvasir	ClinDB	ColDB	ETIS	CVC-T	*Avg*
1	a	No	0.923	0.921	0.789	0.733	0.898	0.853
1	b	No	0.930	0.934	0.821	0.783	0.901	0.873
2	a	No	0.909	0.944	0.806	0.750	0.901	0.862
2	b	No	0.913	**0.947**	0.816	0.783	0.903	0.872
1 + 2	a	No	0.923	0.925	0.794	0.703	0.891	0.847
1 + 2	a	Yes	0.922	0.929	0.808	0.746	0.903	0.862
1 + 2	b	No	**0.931**	0.938	0.821	0.775	0.896	0.872
1 + 2	b	Yes	0.929	0.943	0.822	0.783	0.903	0.876
1 + 2	a + b	No	0.928	0.943	**0.829**	0.779	0.902	0.876
1 + 2	a + b	Yes	0.928	0.945	0.828	**0.791**	**0.905**	**0.879**

**Table 8:** Output values before the sigmoid layer. $P_{i}$ is the *i*-th intermediate prediction mask. Avg_min_ is the average of the minimum values (for each image) before the sigmoid. Avg_max_ is the average of the maximum values (for each image) before the sigmoid. Max_min_ is the max of the minimum values. Min_max_ is the min of the maximum values. $\bar{{\mathrm{Sat}}_{\mathrm{Avg}}}$ is the rate of non-saturated pixels for fusion by the averaging rule. $\bar{{\mathrm{Sat}}_{\mathrm{Sum}}}$ is the rate of non-saturated pixels for fusion by the sum rule. Fusion is among all $P_{i}$s returned by a model.

Method		Min_max_	Avg_max_	Avg_min_	Max_min_	$\bar{{\mathrm{Sat}}_{\mathrm{Avg}}}$	$\bar{{\mathrm{Sat}}_{\mathrm{Sum}}}$
HarDNet-MSEG	*P*	2.51	71.84	−6.53	−11.45	2.57%	2.57%
Polyp-PVT	$P_{1}$	26.22	70.15	−21.06	−30.04	1.62%	1.09%
	$P_{2}$	19.53	38.55	−20.61	−29.47		
HSNet	$P_{1}$	24.21	67.81	−38.49	−69.06	2.92%	0.85%
	$P_{2}$	33.19	87.04	−42.63	−84.26		
	$P_{3}$	31.31	122.01	−63.13	−123.45		
	$P_{4}$	36.47	160.11	−95.23	−165.40		

## Data Availability

All the resources required to replicate our experiments are available at https://github.com/LorisNanni (accessed on 8 May 2023).
